# CoMet: a workflow using contig coverage and composition for binning a metagenomic sample with high precision

**DOI:** 10.1186/s12859-017-1967-3

**Published:** 2017-12-28

**Authors:** Damayanthi Herath, Sen-Lin Tang, Kshitij Tandon, David Ackland, Saman Kumara Halgamuge

**Affiliations:** 1 Department of Mechanical Engineering, The University of Melbourne, Parkville, Melbourne, 3010 Australia; 2 Department of Computer Engineering, University of Peradeniya, Prof. E. O. E. Pereira Mawatha, Peradeniya, 20400 Sri Lanka; 3 Biodiversity Research Center, Academia Sinica, Nan-Kang, Taipei, 11529 Taiwan; 4 Institute of Bioinformatics and Structural Biology, National Tsing Hua University, Hsinchu, 300 Taiwan; 5 Bioinformatics Program, Institute of Information Science, Taiwan International Graduate Program, Academia Sinica, Taipei, 115 Taiwan; 6 Department of Biomedical Engineering, The University of Melbourne, Victoria, 3010 Australia; 7 Research School of Engineering, College of Engineering and Computer Science, The Australian National University, Canberra ACT, 2601 Australia

**Keywords:** Metagenomics, Binning, Contig coverage, Contig composition, DBSCAN algorithm

## Abstract

**Background:**

In metagenomics, the separation of nucleotide sequences belonging to an individual or closely matched populations is termed binning. Binning helps the evaluation of underlying microbial population structure as well as the recovery of individual genomes from a sample of uncultivable microbial organisms. Both supervised and unsupervised learning methods have been employed in binning; however, characterizing a metagenomic sample containing multiple strains remains a significant challenge.

In this study, we designed and implemented a new workflow, Coverage and composition based binning of Metagenomes (CoMet), for binning contigs in a single metagenomic sample. CoMet utilizes coverage values and the compositional features of metagenomic contigs. The binning strategy in CoMet includes the initial grouping of contigs in guanine-cytosine (GC) content-coverage space and refinement of bins in tetranucleotide frequencies space in a purely unsupervised manner. With CoMet, the clustering algorithm DBSCAN is employed for binning contigs. The performances of CoMet were compared against four existing approaches for binning a single metagenomic sample, including MaxBin, Metawatt, MyCC (default) and MyCC (coverage) using multiple datasets including a sample comprised of multiple strains.

**Results:**

Binning methods based on both compositional features and coverages of contigs had higher performances than the method which is based only on compositional features of contigs. CoMet yielded higher or comparable precision in comparison to the existing binning methods on benchmark datasets of varying complexities. MyCC (coverage) had the highest ranking score in F1-score. However, the performances of CoMet were higher than MyCC (coverage) on the dataset containing multiple strains. Furthermore, CoMet recovered contigs of more species and was 18 - 39% higher in precision than the compared existing methods in discriminating species from the sample of multiple strains. CoMet resulted in higher precision than MyCC (default) and MyCC (coverage) on a real metagenome.

**Conclusions:**

The approach proposed with CoMet for binning contigs, improves the precision of binning while characterizing more species in a single metagenomic sample and in a sample containing multiple strains. The F1-scores obtained from different binning strategies vary with different datasets; however, CoMet yields the highest F1-score with a sample comprised of multiple strains.

**Electronic supplementary material:**

The online version of this article (doi:10.1186/s12859-017-1967-3) contains supplementary material, which is available to authorized users.

## Background

Metagenomics has enabled the culture-independent study of the dynamics of microbes in different environments including the human gut [1, 2], soil [3] and seawater surface [4]. Through the analysis of data generated from direct sampling and high-throughput shotgun sequencing of genetic material of microbiota, metagenomics can provide important applications in evaluating the ecology of uncultivable organisms in different habitats [5–7].

Sequence assembly and sequence binning are two key steps involved in a metagenomics experiment. Sequence assembly is performed to generate contigs (i.e. overlapping sequences) from short reads generated in the experiment by identifying the overlapping nucleotide sequences belonging to a particular organism. Sequence binning is the separation of nucleotide sequences belonging to an individual genome or closely related genomes into groups. Binning is mostly adopted as a subsequent step after sequence assembly; however, the possibility of binning before assembling the reads has been suggested to reduce assembly complexity [8].

There are two key metagenomic approaches for taxonomic profiling of a given microbial community: (1) the use of taxonomic barcodes or phylogenetic marker genes, and (2) shotgun sequencing-based approach [9]. The scope of this study is binning datasets obtained using shotgun sequencing. Binning metagenomic sequences is challenging because of the complexities of microbial populations such as variation in abundances and lack of information on genomic sequences of organisms. In addition, the complexities in datasets such as the high volume of data and sequencing/assembly errors make binning a challenging task. Consequently, various binning strategies have been proposed to discriminate nucleotide sequences belonging to species in a metagenomic sample, and have been extensively reviewed (see [10–12]).

Existing binning methods can generally be grouped into taxonomy dependent methods and taxonomy independent methods. Taxonomy dependent methods bin sequences based on reads similarity to known sequences in databases or using supervised learning models (based on reference sequences) (see, for example [13–16]). Taxonomy dependent binning methods are useful in realizing the profile of known organisms in a sample, but are less effective in evaluating microbial populations with unknown species [10]. In contrast, taxonomy independent binning strategies are based on mutual dis-similarities observed in sequences and do not require known sequence data.

Taxonomy independent methods have been shown to be useful in analyzing metagenomic samples that may contain many unknown organisms [17]. Consequently, taxonomy independent strategies which utilize statistical methods for feature extraction, techniques for data visualization and unsupervised learning methods for clustering sequences have been widely adopted for binning [12].

Existing taxonomy independent binning methods may be categorized into two distinct groups based on the features used in them: sequence composition based methods and relative abundance based methods. Sequence composition based approaches utilize the features extracted from nucleotide sequences (or the assembled contigs) of the organisms. Two such compositional features are guanine–cytosine (GC) content and tetranucleotide frequencies. The GC content of a genomic sequence is known to be distinct for various species. For example, it has been shown that GC content is the cause of differences in characteristics such as temperature optimum and tolerance range, and hence is correlated with phylogenetic relationships observed among bacterial populations [18]. Similarly, higher order base composition statistics of the sequences, termed nucleotide frequencies, are considered as species-specific signatures, while tetranucleotide frequencies are used to discriminate species [17, 19–22]. A novel measure of the relative magnitude of biases in base composition, the Oligonucleotide Frequency Derived Error Gradient (OFDEG), has also been proposed and shown to be effective in separating individual genome sequences. Alternatively, the relative abundance of species (or its genomic fragments) has been used as a discriminating feature for binning and is encapsulated by the q-mer frequency of the reads [23, 24] or sequence coverage information [25]. Hybrid binning strategies have been proposed, utilizing both sequence coverage and sequence composition related features [26–28] and/or are based on dis-similarities observed among species, as well as features extracted based on known sequence data [22].

The identification of representative genomic signatures and the use of appropriate clustering methods are important in improving the performances of binning methods. Machine learning methods that are employed in binning have been extensively reviewed [29]. Clustering methods employed in binning methods include agglomerative hierarchical clustering, k-means clustering, k-medoids clustering and model based clustering [29, 30]. However, parameter initialization and specification of the number of bins (k) represent challenges for some existing binning methods [24, 26]. Some clustering methods are prone to outliers, and therefore robust outlier filtering strategies are adopted to improve precision in binning [17]; however, application of robust outlier filtering reduces the total number of contigs being binned [17].

Contig coverage based binning of multiple samples has been suggested before [3]. Furthermore, the use of abundance and genomic composition related features of organisms calculated from multiple metagenomic samples for binning contigs has been recently proposed [29], but the precision of binning methods based on multiple sample data is shown to decrease as the number of samples decreases [27]. A recent approach, namely MyCC [22] has been shown to improve the precision in binning. The use of genomic signatures and marker genes for binning is employed in MyCC workflow and it has been shown to yield higher precision than other binning strategies using compositional and coverage features extracted from multiple metagenomic samples such as CONCOCT and MetaBAT [20–22]. As a binning strategy, MyCC has been shown to be effective for a single metagenomic sample as well; however, binning a sample of multiple strains is shown to be challenging with MyCC [22].

The objective of the present study was to develop a workflow, ‘Coverage and composition based binning of Metagenomes’ (CoMet), to evaluate the use of both contig coverage and compositional features extracted from contigs for binning a single metagenomic sample. CoMet employs unsupervised learning methods so that minimal user inputs are required to cluster contigs. With CoMet, we explored the use of the clustering algorithm, Density-based spatial clustering of applications with noise (DBSCAN) [31] in binning. The advantages of DBSCAN over other clustering methods are that the DBSCAN algorithm handles the outliers effectively, it does not assume a fixed cluster shape and it infers the number of distinct groups from the data automatically.

Furthermore, the coverage values of assemblies are directly correlated to the relative abundances of the organisms in the sample, and hence can be used to discriminate closely related organisms. Compositional features may be similar in closely related species [30] and the use of only compositional features has been shown to result in lower accuracy in samples with contigs from organisms with similar tetranucleotide frequencies [28].

However, most of the existing methods for binning a single metagenomic sample do not consider contig coverage as a primary feature. In contrast, contig coverage has been used as a secondary feature combined with tetranucleotide frequencies in existing methods [20, 22, 32]. Two existing methods that consider both contig coverage and GC content are differential coverage based binning [33] and VizBin [34]. However, differential coverage based binning require data from multiple samples and VizBin require manual selection of bins. CoMet was used to explore the use of contig coverage as a primary feature coupled with GC content for automated binning of a single metagenomic sample and a sample of multiple strains. Furthermore, a set of widely used binning methods and CoMet were evaluated on a set of simulated metagenomes and a real metagenome, considering multiple binning performance measures.

## Methods

### CoMet binning workflow

**Fig. 1:**
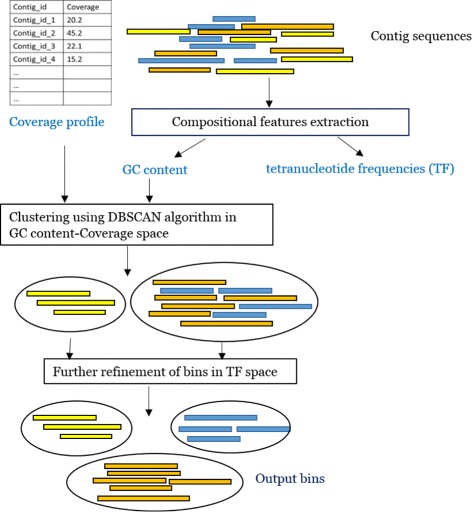
A schematic diagram showing workflow in CoMet. The figure illustrates the key steps involved in proposed binning workflow

CoMet uses contig coverage coupled with contig composition to separate metagenomic contigs into groups of related populations, which may be used to infer the underlying population structure of a microbial sample (Fig. 1). The compositional features of similar genotypes (i.e. strains) may be similar; however, their relative abundances in the sample may differ. Intuitively, the differences in relative abundances of species captured by contig coverage can be used to generate initial groupings. The use of contig coverage has been demonstrated to be effective in improving binning performance [22, 33, 34]. The proposed CoMet workflow consists of three primary steps: (1) compositional feature extraction (2) the primary binning of contigs using DBSCAN algorithm in GC-coverage space, and (3) further refinement of bins considering tetranucleotide frequencies of contigs. These steps are explained in detail in subsequent sections.

#### Compositional features extraction

The compositional features used in CoMet are GC content and tetranucleotide frequencies of the contigs. The inputs to CoMet are nucleotide sequences of the assembled sequences in FASTA format and their coverage values. The compositional features, GC content and tetranuclotide frequencies of the contigs are calculated from sequence data. The input contigs are filtered based on their length (set as 1000 bp in this study) in order to capture a strong representation of the compositional features [17, 32]. The GC content of a contig is calculated as the ratio of guanine + cytosine bases in the contig. The tetranucleotide frequency profile of a contig contains the frequencies of tetramers in a contig. They are computed by scanning the sequence of the contig using one bp sliding window and counting the occurrences of tetramers. The tetranucleotide profile of a contig is computed as the aggregate tetramer frequencies of the contig and its reverse complement, normalised by its total tetramer frequencies.

The coverage profile of the sample ought to be provided. The coverage of a contig is the average number of reads per base from the sample in the contig. The coverage profile is calculated by mapping the assemblies back to reads and maybe extracted from the output of a read alignment tool such as Bowtie 2 [22, 27, 35].

#### Initial clustering using DBSCAN algorithm

With CoMet, initial bins are generated by grouping contigs by considering GC content and coverage. The coverage values are log transformed and the contigs are clustered in GC-log(coverage) space using DBSCAN algorithm. The rationale for this approach is that the coverage values of assemblies are directly correlated to the relative abundances of the organisms in the sample and hence can be used to discriminate closely related organisms. In contrast, compositional features may be similar in closely related species [30]. Contig coverage is coupled with GC content values to have more distinct cluster separations.

The use of the DBSCAN algorithm for binning metagenomic contigs is suggested with CoMet. To the best of our knowledge, DBSCAN algorithm has not previously been applied for binning metagenomic sequences. The DBSCAN algorithm discriminates clusters from noise by identifying densely populated regions with the rationale that the density of points in the same group (i.e. a cluster) must be higher than the points falling outside the group (i.e noise). The primary steps in the DBSCAN algorithm are described in brief next. (See [31] for complete explanation). In DBSCAN, two parameters, *epsilon* and *minimumpoints* are used to distinguish points in a cluster. For a point to be included in a cluster, its neighborhood within a given radius, *epsilon* should contain at least *minimumnumberofpoints* [31]. The parameter *epsilon* refers to the radius of the neighborhood around a point (i.e. *ε*−*neighborhood* of the point). The algorithm begins by selecting an arbitrary data point *c*. If there are more than *minimumpoints* including the point itself, within its *ε*−*neighborhood*, then *c* is marked as a *corepoint* and forms a cluster *C* with the points in its *ε*−*neighborhood*. New points are added to the cluster recursively exploring the *ε*−*neighborhood*s of points in *C* excluding *c*. The process is repeated with a new arbitrarily chosen point when no more points could be added to the cluster *C*. A point belonging to the *ε*−*neighborhood* of a *corepoint*, *x* but with points less than *minimumpoints* in *ε*−*neighborhood* is termed a *borderpoint*. A *borderpoint* get assigned to the cluster that discovers it first. The points that do not get assigned as a *corepoint* or a *borderpoint* are identified as outliers or noise. The implementation of the DBSCAN algorithm, dbscan from the R package fpc [36] was used in our work using *Eucledian* as the distance metric.

Three properties of DBSCAN algorithm are beneficial in alleviating limitations associated with clustering methods used in existing binning approaches such as hierarchical clustering, k-means clustering and finite mixture modeling: (i) the number of clusters does not need to be specified explicitly, (ii) no assumptions about the cluster shape are made, and (iii) outliers can be detected effectively. At the initial coarse clustering step, prior knowledge on similar species may not be given. Therefore, the DBSCAN algorithm was selected over mentioned other clustering methods.

#### Further refinement of bins given tetranucleotide frequencies of contigs

It is assumed that the initial coarse clustering is representative of the underlying population structure, however, the initial coarse groups obtained after the initial clustering step may still contain contigs of multiple species. Therefore, the subsequent refinement of bins in the tetranucleotide space is applied to discriminate contigs of different species that may have been incorrectly grouped into the same group at the initial step. Each cluster that is generated after initial step may be considered as a metagenomic sample of smaller size. The refinement of bins consists of two primary steps. First, the tetranucleotide frequency profiles of the contigs in each cluster are mapped to adequate representations in reduced dimensionality by applying Principal Component Analysis (PCA). Second, contigs in each bin are further clustered using infinite Gaussian mixture modelling with Gibbs sampling [37]. Dimensionality reduction is beneficial when working with high dimensional data to simplify the clustering process while preserving the original feature representation. Since the assumption of normality of the tetranucleotide frequencies distribution has been verified previously [17] the Gaussian mixture modelling was employed.

Many recent binning methods using unsupervised learning methods perform finite Gaussian mixture modeling [17, 27, 38, 39]. A limitation of these finite mixture models-based binning methods is the selection of the number of clusters providing best performance [38]. In CoMet, this need is alleviated by using an infinite Gaussian mixture modeling method namely Dirichlet Process Gaussian Mixture Models (DPGMM) for clustering. DPGMM falls under the class of probabilistic mixture models and can be considered as an extension of finite Gaussian mixture models, removing the need for specification of the number of distinct groups in the dataset.

A finite Gaussian mixture model with *k* components is given by

$$\begin{aligned} &P\left(y|\mu_{1},{\ldots},\mu_{k},\sigma_{1},{\ldots},\sigma_{k},w_{1},{\ldots},w_{k}\right)\\ &= {\sum\nolimits}_{J=1}^{k}\pi_{j}\mathcal{N} \left.\left(\mu_{j},{\sigma_{j}}^{-1}\right) \right) \end{aligned} $$

with the means and inverse variances given by *μ*_*j*_ and *σ*_*j*_ respectively. *w*_*j*_ refers to the mixing weights and $\sum _{j=1}^{k} w_{j}=1$.

An infinite Gaussian mixture model considers a priori *k*→*∞*. A DPGMM is mainly defined by a set of a priori hyper parameters common to all the components and a concentration parameter related to the Dirichlet process (Refer [37] for the complete derivation). Gibbs sampling is a technique commonly used in Monte Carlo simulations to generate samples from complicated multivariate distributions. When generating samples using Gibbs sampling method, the value of a variable is updated based on its conditional distribution given all rest of the variables. Having defined a set of conditional posterior distributions, Gibbs sampling can be used to infer the parameters of a DPGMM using a Markov Chain Monte Carlo (MCMC) approach [37]. Alternatively, a deterministic approach with a variational inference algorithm for Dirichlet Mixture modeling has been suggested [40]. The Selection between variational inference method and Gibbs sampling based MCMC approach is a trade-off between the time and the accuracy. The former is suitable for a fast approximation of the solution while the latter is theoretically guaranteed for accuracy. The implementation of CoMet and evaluations were carried out in R and relevant files are available at https://github.com/damayanthiHerath/comet.

### Comparison with existing binning methods

The use of coverage and compositional features of contigs coupled with unsupervised learning methods for binning a single metagenomic sample is proposed in CoMet. CoMet was evaluated for binning performance along with four methods for binning a single metagenomic sample. They are (1) purely contigs composition based binning method, Metawatt [19], (2) both composition and coverage based binning method, MaxBin [32], (3)a recent binning method based on contig composition and marker genes, MyCC (default) [22], (4) its supplemented version based on contig composition, maker genes and contig coverage, MyCC (coverage) [22]. Both MaxBin and MyCC (coverage) perform clustering of contigs in the combined feature space of contig coverage and compositional features. MaxBin adopts an Expectation Maximization (EM) approach for grouping similar sequences. The clustering algorithm used in MyCC is Affinity Propagation. The implementation of Metawatt was downloaded from https://sourceforge.net/projects/metawatt/. The evaluations of MaxBin were carried out with docker image of MaxBin Version 2.0 accessed from https://downloads.jbei.org/data/microbial_communities/MaxBin/MaxBin.html. The docker image of MyCC downloaded from https://sourceforge.net/projects/sb2nhri/files/MyCC/ and was used for evaluation of MyCC (default) and MyCC (coverage).

### Evaluation on simulated datasets

The binning performances of CoMet and Metawatt, MaxBin, MyCC (default) and MyCC (coverage) were evaluated using four simulated benchmark datasets.

Simulated Illumina sequences of a metagenomic sample comprising 10 genomes have been previously used to benchmark assembly tools [41], and contigs generated by assembling these reads have been used to evaluate binning methods [22]. The reads have been assembled using Ray Meta assembler and coverage profile calculated using Bowtie 2. In this study, mentioned assemblies and the contig coverage values were downloaded from the web resource, https://sourceforge.net/projects/sb2nhri/files/MyCC/Data and were used to evaluate different binning strategies. This dataset is referred as sim10_1.

Two simulated metagenomic datasets of 10 genomes with different relative abundances have been used in evaluation of MaxBin [32]. Generation of 5 million and 20 million Illumina reads from the sample has been simulated using Metasim reads simulator and assemblies have been generated using Velvet assembler [32]. The two sets of assemblies of different overall coverages, 20x and 80x and their coverage profiles were downloaded from https://downloads.jbei.org/data/microbial_communities/MaxBin/MaxBin.html and were used in this study to evaluate different binning strategies. The datasets with overall coverages 20x and 80x are referred as sim10_20x and sim10_80x, respectively.

Binning a metagenomic sample comprised of several closely related species, strains is identified to be a challenging task for existing binning methods [22, 42]. The performances of CoMet and remaining binning methods were evaluated with a metagenomic sample consisting of multiple strains downloaded from CAMI web site. CAMI is a project initiated for creating benchmark datasets of different complexities to evaluate methods for assembly, taxonomic profiling and binning of metagenomics data [42]. Assemblies and abundance profile of a simulated strain dataset comprised of 30 organisms of size 15 Gbp were downloaded from https://data.cami-challenge.org/. Mentioned dataset that was downloaded from CAMI is referred as sim30_cami.

### Evaluation of CoMet on strain datasets with varying coverage distributions

The effect of varying coverage distributions on performances of CoMet was evaluated based on the contigs in sim30_cami dataset which consists of contigs generated from sequences of 30 strains. Random coverage values of the organisms were sampled from 1, 2, 3, 5, 6, 10, 15 and 30 different coverage distributions and their values were in the range of 1–300. For each number of distinct coverage distributions considered, 10 samples were generated with contigs that were assigned coverage values sampled from the given distribution pattern. CoMet was evaluated on the 80 datasets for precision, F1-score and number of species discovered.

### Evaluation on a real metagenome

The metagenomic experiment conducted to analyze human infant gut microbiome [43] was considered for evaluating the applicability of CoMet on real data. The assembled contigs generated from Illumina reads, coverages computed using Bowtie 2 and binning information from the original study were obtained from https://sourceforge.net/projects/sb2nhri/files/MyCC/Data. The outcome of binning of these contigs using CoMet was compared against the results obtained from binning them using MyCC (default) and MyCC(coverage). MyCC (default) and MyCC (coverage) were selected for comparison because they have shown higher performance than other methods in previous work [22]. The experiment has had 18 sequence runs of 11 fecal samples. Since CoMet is suggested for binning a single metagenomic sample, the run with least number of contigs with zero coverages was considered for evaluation.

### Binning performance measures

The true assignments of the contigs (ground truth) are available for the simulated data. For the real metagenome, the binning assignments made in the experiment were downloaded from https://sourceforge.net/projects/sb2nhri/files/MyCC/Data and were used as the gold standards. Based on the gold standards, CoMet and four other binning methods were evaluated using four measures including precision, recall, F1-score and the number of species discovered [22, 23, 27, 32]. The definitions of these measures are provided below. All the binning methods were ranked on their performances in order to make a comprehensive comparison of their performances with different datasets.

Assume there are *N* genomes in the dataset and the method outputs *M* clusters *C*_*i*_ (1≤*i*≤*M*). Let *R*_*ij*_ be the number of reads in *C*_*i*_ which are from genome *j* and *C*_*j*_ represent genome *j* when *R*_*ij*_=*max*_*j*_ *R*_*ij*_. The overall precision, recall and F1-score are calculated as below.

1 $$ Precision (\%) = {\frac{\sum_{i=1}^{M} max_{j} R_{ij}}{\sum_{i=1}^{M} \sum_{j=1}^{N} R_{ij}}}*100  $$

2 $$ \begin{aligned} Recall (\%)= {\frac{\sum_{j=1}^{N} max_{i} R_{ij}}{\sum_{i=1}^{M} \sum_{j=1}^{N} R_{ij} + number\ of\ unclassified\ reads}}*100 \end{aligned}  $$

F1-score is the harmonic mean of precision and recall and is defined as

3 $$ F1 = 2* \frac{Precision*Recall}{Precision+Recall}  $$

Given all contigs originated from a particular genome *S*, if there is a cluster *C* such that > 50*%* contigs in *C* belongs to *S* and > 50*%* of the contigs of *S* are in bin *C*, then the *S* genome is considered to be discovered by the bin *C*. The total number of discovered species with each dataset is then calculated accordingly.

## Results and discussion

### Binning performance comparison of different binning strategies

The binning strategies based on both contig coverage and compositional features (MaxBin, MyCC (default), MyCC (coverage), CoMet) yielded higher precision than binning using only tetranucleotide frequencies of contigs (Metawatt) (Table 1). CoMet had the highest ranking score in precision, followed by MyCC (coverage), MyCC (default) and MaxBin. The relative abundances of genomes considered in sim10_1 are similar [22, 41]. The precisions yielded from Metawatt and CoMet, MyCC (default) and MyCC (coverage) on this sample of genomes with similar abundances are comparable and are in the range of 97–98%.

**Table 1:** Precision comparison between CoMet and other contig coverage and/or composition based binning methods

Dataset	Metawatt	MaxBin	MyCC (default)	MyCC (coverage)	CoMet
sim10_1	96.69 (4)	92.44 (5)	97.47 (2)	97.42 (3)	**97.94 (1)**
sim10_20x	84.25 (5)	96.90 (2)	90.66 (4)	96.71 (3)	**98.66 (1)**
sim10_80x	95.13 (5)	95.63 (4)	98.55 (2)	**98.63 (1)**	97.12 (3)
sim30_CAMI	53.68 (5)	66.60 (4)	75.02 (2)	75.02 (2)	**92.93 (1)**

Binning methods are ranked based on their precision with different datasets with their ranks given in parentheses. Bold values indicate the highest of the precisions

The relative abundances of genomes in sim10_20x and sim10_80x are different. All the binning methods yielded similar precisions on the sample which consists of genomes of different relative abundances and high coverage (sim10_80x). However, on sim10_20x which has lower coverage than sim10_80x, binning methods based on both contig coverage and composition provided higher precisions than the binning method based only on contig composition. From the precisions obtained with sim10_20x and sim10_80x, it is observed that when applied on two samples of different overall contig coverages, CoMet and MaxBin yield higher precisions with the low coverage sample than with the high coverage sample.

The precision of CoMet was significantly higher than the other binning approaches when applied to the strain dataset comprised of 30 organisms. Multiple strains may have similar compositional features and hence, it may be difficult to discriminate them by only considering their genetic composition; however, their relative abundances in the sample which can be inferred from their contigs coverage may be different. Consequently, the proposed approach of binning may be beneficial in discriminating species from a metagenomic sample of multiple strains.

CoMet was higher in binning precision than MaxBin. MaxBin considers both tetranucleotide frequencies and coverage values in a single feature space. On the contrary, CoMet adopts a two-tired approach considering contig coverage and tetranucleotide frequencies separately, and was shown to improve precision over MaxBin.

In comparison to MyCC (default) and MyCC (coverage), CoMet yielded higher or comparable binning precisions. MyCC primarily uses k-mer frequencies of contigs in clustering and marker genes for cluster correction. In MyCC (coverage), contig coverage is considered in addition to the k-mer frequencies for clustering contigs. The results from MyCC and CoMet show that, the integration of coverage in conjunction with compositional features did not yield an improvement in precision over MyCC (default) except with sim10_20x sample. However, the precision improvement of CoMet over MyCC(default) is higher than the precision improvement of MyCC(coverage) over MyCC (default). These results suggest that, a tiered binning approach may yield higher precisions than binning contigs in a single feature space.

Binning strategies were evaluated on their recall in binning datasets of different complexities (in Additional file 1: Table S1). Both MaxBin and MyCC (coverage) had the highest ranking score in recall, while Metawatt had the lowest ranking score in recall. CoMet had a lower ranking score than MaxBin, MyCC (coverage) and MyCC (default), but yielded higher or comparable recall values in comparison to Metawatt. In CoMet, a set of contigs is filtered out if they act as outliers in the initial binning step or belong to an output bin of smaller size. Consequently, a set of input contigs remains unclassified which leads to the lower recall. Moreover, multiple bins representing a single species lowers the recall. MyCC (coverage) improves the recall of MyCC (default) except on the contigs from genomes of similar abundances (sim10_1).

The binning strategies considered in this work, vary in their performances in F1-score (Table 2). Considering the ranking scores in F1-score, MyCC (coverage) had the highest ranking score followed by MyCC (default), MaxBin, CoMet and Metwatt. It suggests that, the binning approach in MyCC is useful in improving the F1-score. CoMet had the lowest F1-score on the dataset of genomes of similar abundances (sim_10x) in comparison to its F1-scores on other datasets. As far as the contigs of genomes with different relative abundances are considered (i.e. sim10_20x and sim10_80x), F1-scores of both CoMet and MaxBin were higher on the low coverage dataset than that on the high coverage dataset. In contrast, the F1-scores obtained from MyCC (default) and MyCC (coverage) on low coverage dataset (sim10_20x) were lower than that on the high coverage dataset (sim10_80x). CoMet yielded highest F1-score on contigs of multiple strains; however, the F1-scores of all the binning methods on strain dataset are lower than their F1-scores on other datasets.

**Table 2:** F1-Score comparison between CoMet and other contig coverage and/or composition based binning methods

Dataset	Metawatt	MaxBin	MyCC (default)	MyCC (coverage)	CoMet
sim10_1	91.58 (4)	94.91 (3)	**97.47 (1)**	96.09 (2)	89.28 (5)
sim10_20x	70.5 (5)	**98.16 (1)**	83.46 (4)	88.35 (3)	96.7 (2)
sim10_80x	75.21 (5)	95.61 (3)	98.55 (2)	**98.64 (1)**	88.56 (4)
sim30_CAMI	60.55 (5)	75.58 (4)	80.93 (2)	80.93 (2)	**82.71 (1)**

Binning methods are ranked based on their F1-score with different datasets with their rank given in parentheses. Bold values indicate the highest of the F1-scores

Furthermore, CoMet and existing contig coverage and/or composition based binning methods were evaluated on the number of species identified (Table 3). MyCC (default) and MyCC (coverage) discovered the highest number of species from the sim10_1 dataset. Considering sim10_20x and sim10_80x, all binning methods recovered more species from the high coverage sample (sim10_80x) than from the low coverage sample. Moreover, both CoMet and Metawatt identified the highest number of species from the low coverage sample (sim10_20x). The results show that CoMet was able to recover 40–90% of the species in a sample. Furthermore, CoMet identified the highest number of species from the dataset of multiple strains. MyCC (default) and MyCC (coverage) ranks second in number of species identified from the strain dataset.

**Table 3:** The number of species recovered from different binning approaches

Dataset	Metawatt	MaxBin	MyCC (default)	MyCC (coverage)	CoMet
sim10_1	9 (3)	8 (5)	**10 (1)**	**10 (1)**	9 (3)
sim10_20x	**4 (1)**	3 (3)	3 (3)	2 (5)	**4 (1)**
sim10_80x	6 (5)	**10 (1)**	**10 (1)**	**10 (1)**	7 (4)
sim30_CAMI	9 (5)	13 (4)	18 (2)	18 (2)	**20 (1)**

Binning methods are ranked based on number of species discovered with their rank given in parentheses. Bold values indicate the highest of the number of species identified

The GC content distributions of the datasets considered in this study have been of arbitrary form (in Additional file 1: Figure S1) and are skewed to the left in all datasets except sim10_1 (in Additional file 1: Figure S1). The GC content values of the contigs in the datasets were in the range of 12–86. The GC content distribution of the contigs in sim30_CAMI datasets is the most left skewed distribution because most of the species in the dataset had higher and similar GC contents. The precision of CoMet with sim30_CAMI was lower than the precision of CoMet with other datasets. CoMet may be used to analyze contigs of different GC content distributions. Similar to other binning approaches, CoMet perform better on samples with species with distinct compositional features.

DBSCAN algorithm can extract clusters of different shapes, but will be hindered by the existence of clusters of different densities [44]. The GC-log(coverage) distributions of the contigs in the datasets considered in this study demonstrates the applicability of the DBSCAN algorithm for clustering contigs in GC-log(coverage) space (in Additional file 1: Figure S2–S5). The clusters in the GC-log(coverage) space do not have substantial differences in densities, and the number of distinct components cannot be determined without a prior knowledge of the datasets. Therefore, DBSCAN algorithm may be considered the most appropriate algorithm for the initial coarse clustering of the contigs.

### Binning a real metagenome using CoMet

With the contigs from the metagenome of infant gut microbiome, CoMet resulted in a precision of 71% and an F1score of 67%. These results were compared against MyCC (default) and MyCC (coverage) which have been shown to have better performances than other binning methods before [22]. MyCC (default) and MyCC (coverage) both resulted in a precision of 36% and an F1score of 49%. The number of species discovered from CoMet, MyCC (default) and MyCC (coverage) was 6.

### Binning performance with strain dataset from CAMI

CoMet was shown to be effective in binning the metagenomic sample of multiple strains (sim30_CAMI) with the highest precision associated with the highest number of species identified. The percentages of species identified from the strain dataset using CoMet, MyCC (default) and MyCC (coverage) were 66, 60 and 60 respectively. Furthermore, for all the identified species from each binning method, the precision in binning contigs from each species and percentage of contigs binned from each species were calculated (Table 4).

**Table 4:** Individual precision and contigs binned from each identified species from the strain dataset from CAMI

Taxon Id	MaxBin		Metawatt		MyCC (default)		MyCC (coverage)		CoMet	
	Precision	Contigs binned (%)	Precision	Contigs binned (%)	Precision	Contigs binned (%)	Precision	Contigs binned (%)	Precision	Contigs binned (%)
1	NI	NI	NI	NI	NI	NI	NI	NI	**78.33**	**94.63**
2	96.15	**100**	NI	NI	NI	NI	NI	NI	**100**	88
3	NI	NI	93.16	63.08	93.58	87.06	93.58	87.06	**96.04**	**92.23**
4	88.74	87.31	NI	NI	89.37	**98.81**	89.37	**98.81**	**100**	51.32
5	NI	NI	74.84	56.93	97.94	**87.58**	97.94	**87.58**	**100**	67.61
6	NI	NI	61.84	58.02	83.82	77.03	83.82	77.03	**100**	77.03
7	NI	NI	96.22	59.89	96.59	**90.87**	96.59	**90.87**	**98.31**	58.17
8	NI	NI	NI	NI	NI	NI	NI	NI	**98.33**	96.72
9	55.18	86.26	NI	NI	93.46	**88.32**	93.46	**88.32**	**99.73**	50.07
10	NI	NI	80.72	57.41	**98.21**	**77.37**	**98.21**	**77.37**	NI	NI
11	92.96	**96.54**	NI	NI	68.18	51.92	68.18	51.92	**100**	51.15
12	97.25	**96.66**	83.25	92.29	96.1	89.97	96.1	89.97	**100**	63.94
13	50	**87.5**	NI	NI	52.17	75	52.17	75	**100**	75
14	51.14	63.38	NI	NI	65.22	63.38	65.22	63.38	**88.89**	**67.61**
15	53.3	57.74	NI	NI	82.29	**85.71**	82.29	**85.71**	**98.13**	62.13
16	NI	NI	NI	NI	NI	NI	NI	NI	**100**	**62.5**
17	NI	NI	NI	NI	**78.14**	50.55	**78.14**	50.55	51.09	**97.92**
18	72.58	**93.75**	NI	NI	66.1	81.25	66.1	81.25	**95.56**	89.58
19	NI	NI	NI	NI	NI	NI	NI	NI	**100**	**58.39**
20	75.05	94.62	82.2	84.17	92.54	**96.77**	92.54	**96.77**	**100**	96.24
20	73.25	74.78	NI	NI	**77.09**	94.93	**77.09**	94.93	NI	NI
22	88.72	87.41	85.23	64.13	**89.79**	78.15	**89.79**	78.15	NI	NI
23	93.97	99	84.29	86.51	96.41	**98.66**	96.41	**98.66**	**100**	70.57

Bold data represent the highest precisions and highest percentage of contigs binned for each identified species. NI: Not Identified

CoMet was able to discover 20 species while MaxBin, MyCC, MyCC (coverage) and Metawatt discovered 13, 18, 18 and 9 species, respectively. The average percentage of contigs binned using CoMet was 73.5, while the average percentage of contigs binned using Metawatt and MaxBin were 69.1 and 86.5, respectively. In addition, the average percentage of contigs binned using MyCC (default) and MyCC (coverage) was 81.85. In comparison to the other binning methods, the precision of recovering individual species of CoMet was higher. However, the percentage contigs binned using CoMet ranked lower compared to that using the other binning methods considered in this study.

CoMet identified 4 species that were not identified by any of the other binning methods with 94.2% average precision. The number of species that has not been identified by CoMet, but has been able to be identified using any remaining binning method is one. The results also show that CoMet and MyCC are complementary in terms of precision in recovering individual strains. In the cases where a given strain was not identified using CoMet or was identified with lower precision using CoMet, MyCC has identified that strain with the highest precisioin and vice versa. However, CoMet yielded 95.2% average precision, whereas, with MyCC, the average precision was 84.3%. In summary, the results show that CoMet is able to discriminate many species with high precision from a sample of multiple strains which is confronting for the other binning methods.

**Fig. 2:**
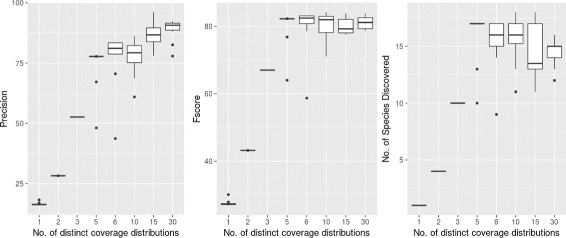
Performance of CoMet on contigs with different number of distinct coverage distributions. The figure shows the variations of binning performances of CoMet as the differences in contig coverage values of a sample of multiple strains vary

CoMet was evaluated further on 80 strain datasets generated based on sim30_cami (Fig. 2). When all the contigs in the sample have similar coverage values and are similar in composition, the precision in binning is the lowest. The precision in binning has improved as the number of distinct coverage distributions increases. F1score and the number of species discovered are higher in samples with more distinct number of coverage distributions (5,6,10,15 and 30) than in samples with less distinct number of coverage distributions (1,2,3).

## Conclusions

In the present study, we proposed CoMet for binning contigs in a metagenomic sample. Both contig coverage and composition are utilized in CoMet to discriminate contigs belonging to similar genotypes. Employing unsupervised learning methods for grouping contigs, CoMet was implemented to be executed with minimal user inputs. In CoMet workflow, contigs are grouped in two steps, first considering their GC content values and coverages, and second given their tetranucleotide frequencies. In order to remove the outliers effectively and learn the number of distinct groups automatically, the DBSCAN algorithm is employed in the first step.

An assembly step is not included in CoMet, therefore sequence assembly should be performed before analyzing sequence data using CoMet. The outcomes of CoMet are independent of the assembly method and it is assumed that assembly of sequences and computation of coverage profile is performed with high accuracy. The datasets considered in this study have been generated using different assemblers and no bias was incurred on the evaluation of different binning methods.

CoMet demonstrated higher precision than a binning method based only on contig composition. Moreover, it yielded higher or comparable precision in comparison to other binning methods that consider both contig coverage and contig composition. Furthermore, CoMet showed a significant improvement in precision in binning of a metagenomic sample consists of multiple strains. The variation in the relative abundances of genomes in a sample is beneficial in binning contigs with similar compositional features and is exploited by CoMet by using contig coverage in its work flow. The precision in binning with CoMet is demonstrated to increase as the distinction in coverage distribution of the organisms in the sample increases.

The simulated datasets considered in this study represent different microbial communities and experimental setups. The evaluations in our study show that performances of different binning strategies vary depending on the nature of the sample. CoMet was ranked first or second in the number of species discovered. Different binning strategies were associated with varying F1-scores on different datasets. CoMet was significantly higher in F1-score than the other binning methods on the strain dataset. All the binning methods considered in this study are shown to be complementary to each other in F1-scores and their performances in discovering individual species. CoMet ranks lower in recall compared to the other binning methods. Further work may be carried out to improve the recall yielded from CoMet, including devising an effective method for assigning the unclassified contigs into bins identified with high precision, merging or splitting of bins, and evaluation of overall binning performance.

As demonstrated with the datasets considered in this study, CoMet can analyze contigs forming clusters with similar densities in GC-log(coverage) space with higher precision. Extending CoMet to be applicable on contigs with significant differences in their range of GC contents and coverages (hence forming clusters of different densities), ought to be considered in future research.

The results of our study suggest that binning samples of multiple strains is a challenging task. However, the benchmark data available for evaluating binning methods on samples of multiple strains is limited. Therefore, future work on the design and development of benchmark datasets similar to CAMI is beneficial in improving the robustness of binning methods. Such datasets may represent recent experimental setups and different compositions of microbial communities including samples of multiple strains.

CoMet utilizes combinations of features of genomic sequences and adopts a purely unsupervised approach. Appropriate clustering methods are employed in CoMet in order to bin contigs with high precision and in an automated manner. The results of this study confirm that both the identification of suitable feature representations and clustering methods is important in improving the precision in characterizing metagenomic samples with various compositions of organisms in an automated and a database-independent manner.

## Additional file

Additional file 1 Supplementary Material file contains the details of recall values obtained in this study and the GC content distributions and the GC content - log(Coverage) distributions of the contigs in the simulated datasets considered in this study. (PDF 203 kb)

## Abbreviations

CoMet: Coverage and composition based binning of Metagenomes
DBSCAN: Density-based spatial clustering of applications with noise
