# Supplementary material for: CoMet: a workflow using contig coverage and composition for binning a metagenomic sample with high precision
Source: BMC Bioinformatics. 2017 Dec 28;18(Suppl 16):571. doi: 10.1186/s12859-017-1967-3 (PMC5751405; doi:10.1186/s12859-017-1967-3)
Supplement: Supplementary file 1 — Supplementary Material file contains the details of recall values obtained in this study and the GC content distributions and the GC content - log(Coverage) distributions of the contigs in the simulated datasets considered in this study. (PDF 203 kb) [file 12859_2017_1967_MOESM1_ESM.pdf]

Supplementary Material

Table S1: Recall comparison between CoMet and other contig coverage and/or contig composition based binning methods

Dataset	MetaWatt	MaxBin	MyCC (default)	MyCC (coverage)	CoMet
sim10_1	86.98(4)	**97.51 (1)**	97.47 (2)	94.79 (3)	82.02 (5)
sim10_20x	60.61 (5)	**99.45 (1)**	77.32 (4)	81.33 (3)	94.81 (2)
sim10_80x	62.19 (5)	95.58 (3)	98.55 (2)	**98.64 (1)**	81.38 (4)
sim30_CAMI	69.42 (5)	87.35 (3)	**87.86 (1)**	**87.86 (1)**	74.51 (4)

Recall values obtained using different binning methodologies. Binning methods are ranked based on their recall with different datasets with their rank given in parentheses. Bold values indicate the highest of the recall values

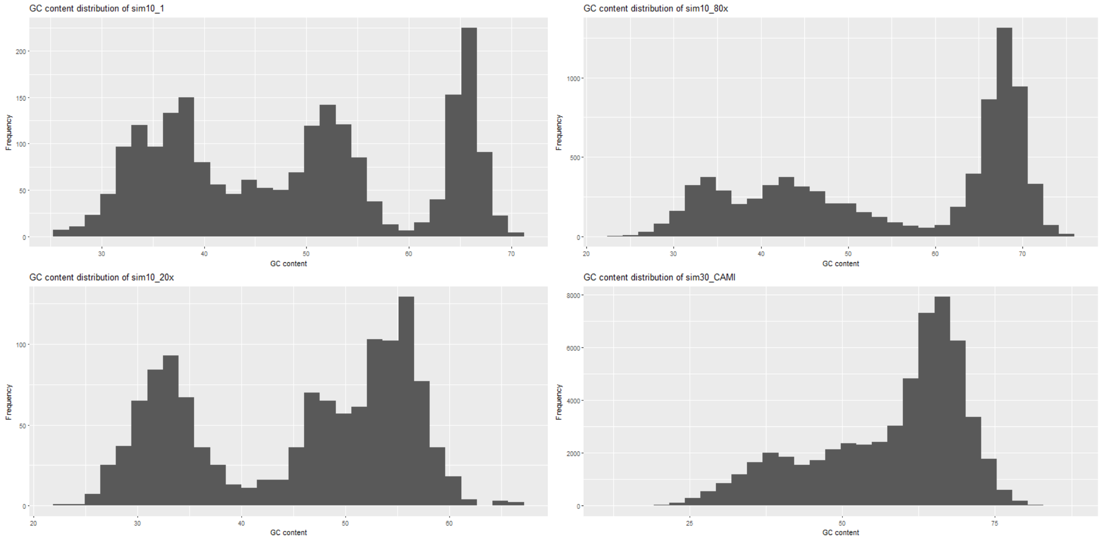

Figure S1: GC content distributions of the contigs in the simulated datasets considered in the study

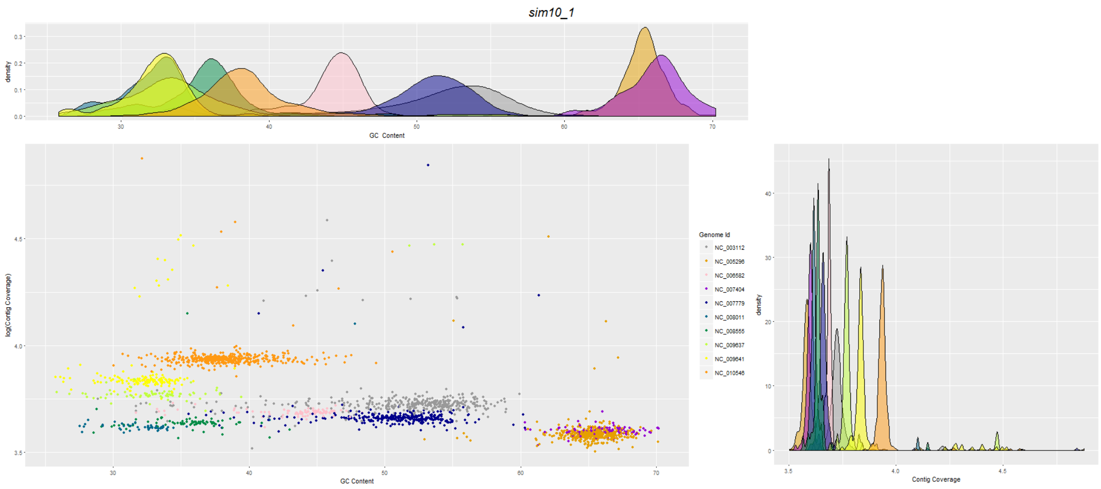

Figure S2: GC-log(coverage) distribution of sim10_1 dataset

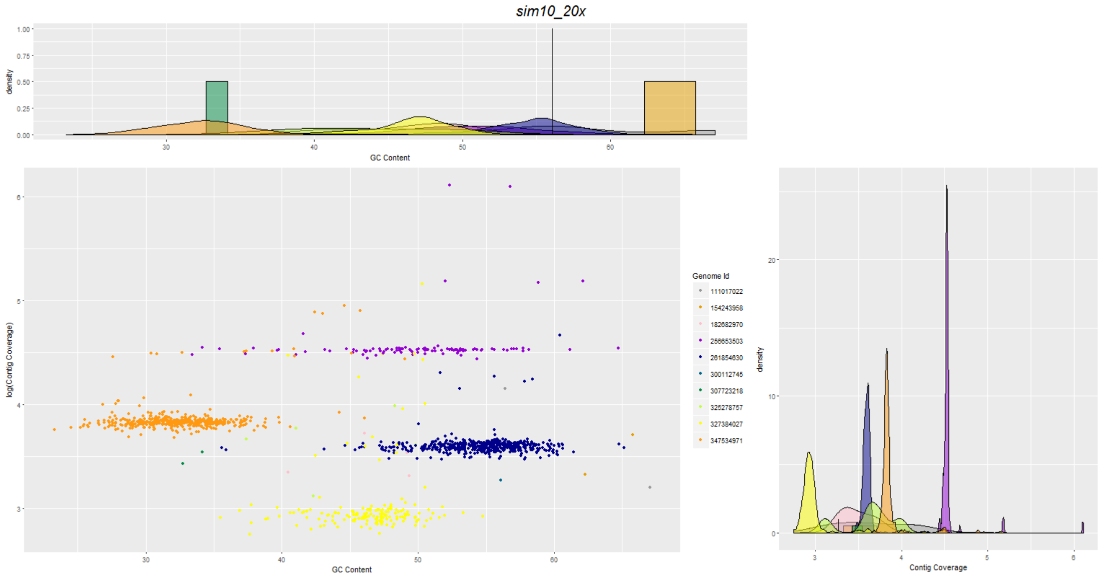

Figure S3: GC-log(coverage) distribution of sim10_20x dataset

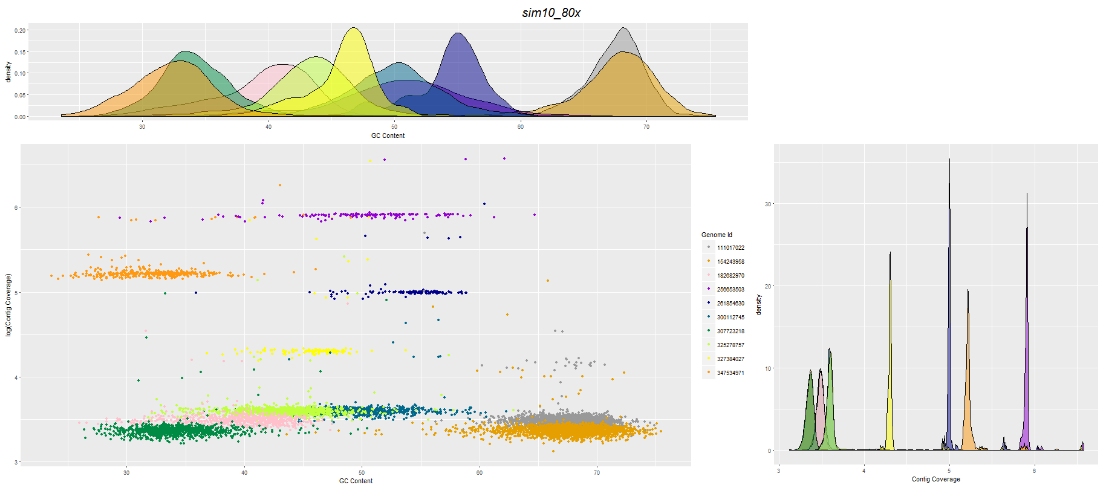

Figure S4: GC-log(coverage) distribution of sim10_80x dataset

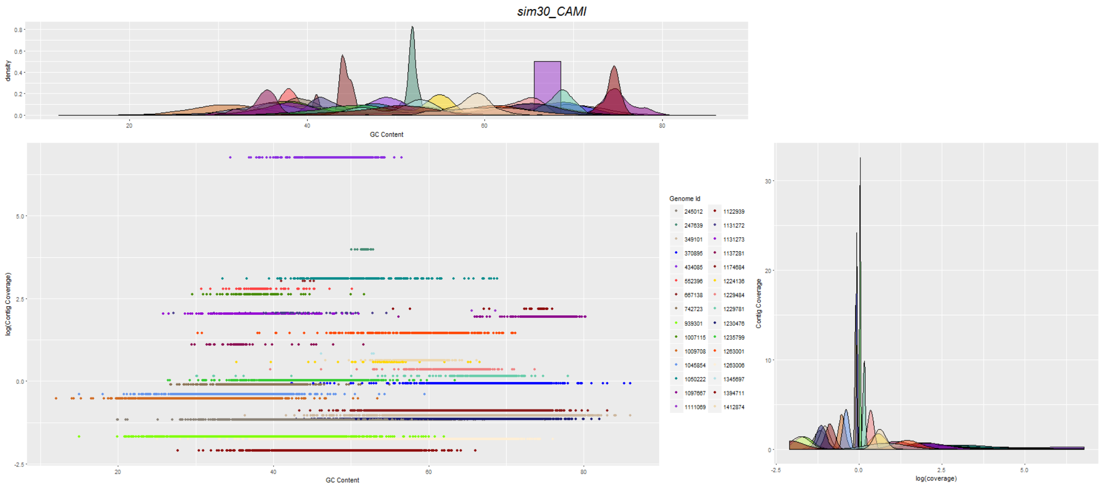

Figure S5: GC-log(coverage) distribution of sim30_CAMI dataset
